# Post-illumination activity of SnO_2_ nanoparticle-decorated Cu_2_O nanocubes by H_2_O_2_ production in dark from photocatalytic “memory”

**DOI:** 10.1038/srep20878

**Published:** 2016-02-16

**Authors:** Lingmei Liu, Wuzhu Sun, Weiyi Yang, Qi Li, Jian Ku Shang

**Affiliations:** 1 Environment Functional Materials Division, Shenyang National Laboratory for Materials Science, Institute of Metal Research, Chinese Academy of Sciences, Shenyang 110016, P. R. China; 2 Department of Materials Science and Engineering, University of Illinois at Urbana-Champaign, Urbana, Illinois 61801, USA

## Abstract

Most photocatalysts only function under illumination, while many potential applications require continuous activities in dark. Thus, novel photocatalysts should be developed, which could store part of their photoactivity in “memory” under illumination and then be active from this “memory” after the illumination is turned off for an extended period of time. Here a novel composite photocatalyst of SnO_2_ nanoparticle-decorated Cu_2_O nanocubes is developed. Their large conduction band potential difference and the inner electrostatic field formed in the *p*-*n* heterojunction provide a strong driving force for photogenerated electrons to move from Cu_2_O to SnO_2_ under visible light illumination, which could then be released to react with O_2_ in dark to produce H_2_O_2_ for its post-illumination activity. This work demonstrates that the selection of decoration components for photocatalysts with the post-illumination photocatalytic “memory” could be largely expanded to semiconductors with conduction band potentials less positive than the two-electron reduction potential of O_2_.

Over the past a few decades, semiconductor-based photocatalysts have been widely explored for both solar energy conversion and environmental applications^1^,^2^,^3^,^4^,^5^. It is generally recognized that various reactive oxygen species (ROSs) could be produced by photocatalysts *in situ* under proper illumination to disinfect microorganisms and degrade organic pollutants at ambient temperature and pressure^6^,^7^,^8^,^9^. Most of these photocatalysts only functioned under illumination because their production of ROSs relied on continuous illumination to generate electron-hole pairs^10^,^11^. However, many potential applications require the continuous activity in the dark for an extended period of time. For example, nosocomial infection by microorganism transmit in hospitals is among the top death causes in many countries, which could be controlled by creating self-disinfection environment in hospitals^12^,^13^. Thus, if a photocatalyst could store part of its photoactivity in “memory” under visible light illumination and then be active from this “memory” after the illumination is turned off for an extended period of time, it could provide continuous solar-powered disinfection during daytime and at night to save lives with a high efficiency and relatively low cost/energy consumption.

Recently, an interesting post-illumination photocatalytic “memory” effect was found in several photocatalytic material systems, which could be active in the dark after the illumination was switched off^14^,^15^,^16^,^17^,^18^,^19^. For single-crystalline Se nanorods^17^ and semimetal Bi nanoparticles^18^, a few charge carriers were found to remain at their surfaces and participate in the ^•^OH production for their activity after the cease of illumination, and their activity in the dark could last no longer than 0.5 h due to the very limited charge carrier amount. For TiON/PdO^14^,^15^, Cu_2_O-NS/TiO_2_-NI^16^, and I-TiO_2_^19^, however, photogenerated electrons could transfer from the light absorber component to the decoration component, be trapped, and then be released to react with O_2_ in the environment to produce ^•^O_2_^−^ when the light illumination was turned off. ^•^O_2_^−^ could subsequently react with H_2_O to produce ^•^OH. Due to the gradual release of trapped photogenerated electrons, their activity in dark could last for more than 10 h, which is desirable for the construction of continuous solar-powered photocatalytic disinfection/degradation systems effective for both daytime and at night.

It is generally believed that the redox ability of photogenerated electrons and holes highly relied on the conduction and valence band potentials of the photocatalyst^20^. The one-, two-, and four-electron reduction potentials of O_2_ could be expressed as reactions (1) to (3) as following^8^:


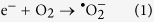



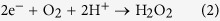



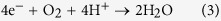


Till now, photocatalysts with the post-illumination photocatalytic “memory” effect required that photogenerated electrons were trapped on decoration components with the conduction band potential negative than the one-electron reduction potential of O_2_ (−0.05 V vs NHE (Normal Hydrogen Electrode)) to react with O_2_ in the dark to produce ^•^O_2_^−^ and subsequently ^•^OH, which largely limited the selection of decoration components. Their relatively more negative conduction band potentials also lowered the potential difference between their conduction bands and that of the light absorber components. Thus, it would be interesting to examine if a decoration component with the conduction band potential less positive than the two-electron reduction potential of O_2_ (0.68 V vs NHE) could be effective to generate activity from the production of H_2_O_2_ in the dark, which could not only largely expand the the selection of potential decoration components but also increase the conduction band potential difference to enhance the driving force for the photogenerated electrons to be injected from the light absorber component’s conduction band to that of the decoration component for their better transfer, trapping and subsequent release.

As an *n*-type, wide band gap semiconductor with interesting chemical, physical and mechanical properties, tin dioxide (SnO_2_) had been extensively studied for applications in gas sensors, dye-based solar cells, transparent conducting electrodes, and catalyst supports^21^,^22^. The chemical state of Sn could exchange between Sn^2+^and Sn^4+^by trapping and release electrons, while it has a conduction band potential (0.4 V vs NHE) less positive than the two-electron reduction potential of O_2_^22^,^23^. Thus, it could have the potential to serve as the decoration component in a composite photocatalyst system to trap the photogenerated electrons injected from the light absorber component, and release them in the dark by the reaction with O_2_ to produce active H_2_O_2_ to possess the post-illumination photocatalytic “memory” effect. In this work, we designed a novel Cu_2_O/SnO_2_ composite photocatalyst composed of Cu_2_O nanocubes decorated with SnO_2_ nanoparticles (Cu_2_O/SnO_2_), in which Cu_2_O nanocubes served as the main light absorption component for a good visible light absorption capability while SnO_2_ nanoparticles formed *p*-*n* heterojunctions of good contact with Cu_2_O nanocubes to serve as the decoration component. The large potential difference (~1.5 eV) between the conduction bands of Cu_2_O and SnO_2_^23^, combined with the inner electrostatic field ξ formed in the *p*-*n* heterojunction, provided a strong driving force for the photogenerated electrons to move from Cu_2_O to SnO_2_ through the heterojunction, which resulted in the enhanced photocatalytic performance under visible light illumination from better charge-carrier separation. The post-illumination photocatalytic “memory” effect was observed as expected for this composite Cu_2_O/SnO_2_ photocatalyst, and the working mechanism was verified as the production of H_2_O_2_ by the release of trapped photogenerated electrons from SnO_2_ to react with O_2_ in the dark.

## Results

### The formation and morphology of SnO_2_ nanoparticle-decorated Cu_2_O nanocubes

**Figure 1:**
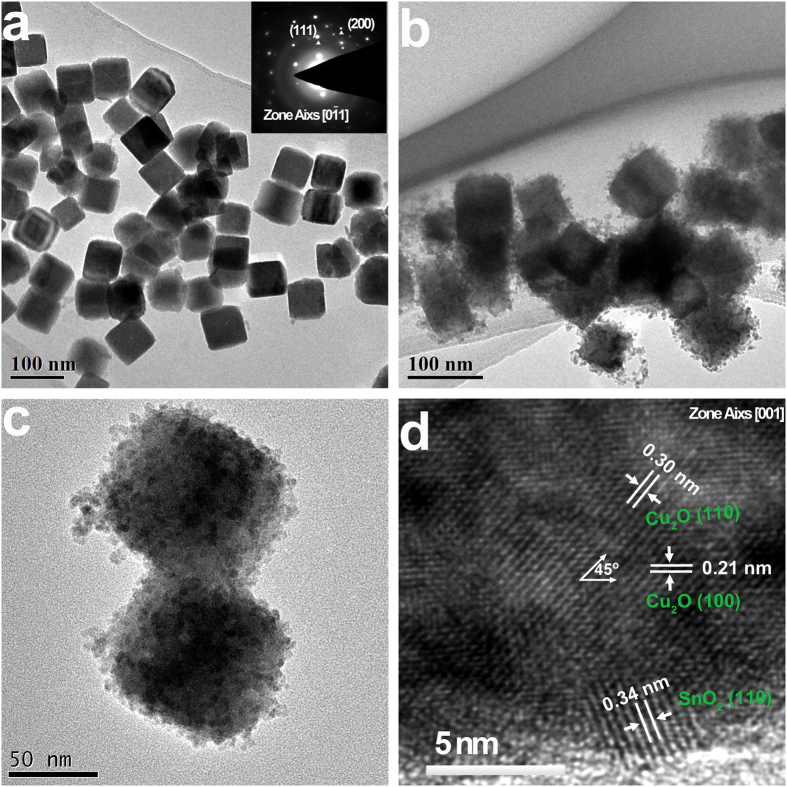
(**a**) The TEM image of the as-prepared Cu_2_O sample. (Note, insert plot in Fig. 1a shows its corresponding selected area electron diffraction pattern.) (**b**) and (**c**) TEM images of Cu_**2**_O nanocubes decorated with SnO_2_ nanoparticles. (**d**) HRTEM image of the Cu_2_O/SnO_2_ interface area.

The electron-hole pair recombination could be largely reduced in single crystal photocatalysts because they have much fewer defects compared with their polycrystalline counterparts, where the electron-hole pair recombination tends to occur^24^. Figure 1a shows the TEM image of the as-prepared Cu_2_O sample and the insert in Fig. 1a shows the corresponding selected area electron diffraction (SAED) pattern. It clearly demonstrated that the sample was composed of desirable single crystal nanocubes, which would favor the transportation of photogenerated electrons/holes and was ideal for constructing heterojunctions with SnO_2_ nanoparticles in our material design. The average edge length of these Cu_2_O nanocubes was ~70 nm, and all their six exposed surfaces were {100} facets. Their fine nanosize could largely increase their specific surface area compared with their counterparts with submicron sizes^25^, beneficial to their contact efficiency with pollutants in water.

The control of the hydrolytic speed of tin precursors was critical for the formation of uniformly dispersed SnO_2_ nanoparticles on these Cu_2_O nanocubes. In our approach, ethyl acetate (C_4_H_8_O_2_) was chosen as the hydrolysis agent to get a good dispersion of SnO_2_ nanoparticles onto the Cu_2_O nanocube surface. As shown in Fig. 1b, the Cu_2_O nanocube morphology was well preserved after the deposition and subsequent hydrothermal process to decorate SnO_2_ nanoparticles onto the Cu_2_O nanocube surface and their crystallization. The surfaces of Cu_2_O nanocubes became relatively rough after the SnO_2_ nanoparticle decoration. Figure 1c shows the TEM image of SnO_2_ nanoparticle-decorated Cu_2_O nanocubes with a higher magnification. It demonstrated clearly that fine SnO_2_ nanoparticles distributed uniformly on surfaces of Cu_2_O nanocubes, and their average size was ~5 nm.

Figure 1d shows a representative HRTEM image of the Cu_2_O/SnO_2_ interface area on these SnO_2_ nanoparticle-decorated Cu_2_O nanocubes. The HRTEM image of the SnO_2_ nanoparticle area verified their highly crystallized structure. One set of lattice planes could be clearly observed with the *d*-spacing at ~0.34 nm, corresponding to the (101) plane of the tetragonal rutile structure of SnO_2_ phase. The HRTEM image of the Cu_2_O nanocube area also verified its highly crystallized structure. The electron beam was aligned along [001] direction, two sets of lattice planes could be clearly observed with the *d*-spacing at ~0.30 nm and ~0.21 nm, respectively, and their separation angle was ~45^o^, corresponding to the (110) and (100) planes of the fcc Cu_2_O phase. The good crystallization of both Cu_2_O nanocubes and SnO_2_ nanoparticles was beneficial to a good photocatalytic performance due to their lack of defects. The observed Cu_2_O/SnO_2_ interface indicated that these SnO_2_ nanoparticles grew on Cu_2_O nanocubes through our synthesis approach and *p*-*n* heterojunctions were formed with good contact between *p*-type Cu_2_O and *n*-type SnO_2_, beneficial to the photoexcited electron transfer between them.

### Crystal structure and chemical composition of SnO_2_ nanoparticle-decorated Cu_2_O nanocubes

**Figure 2:**
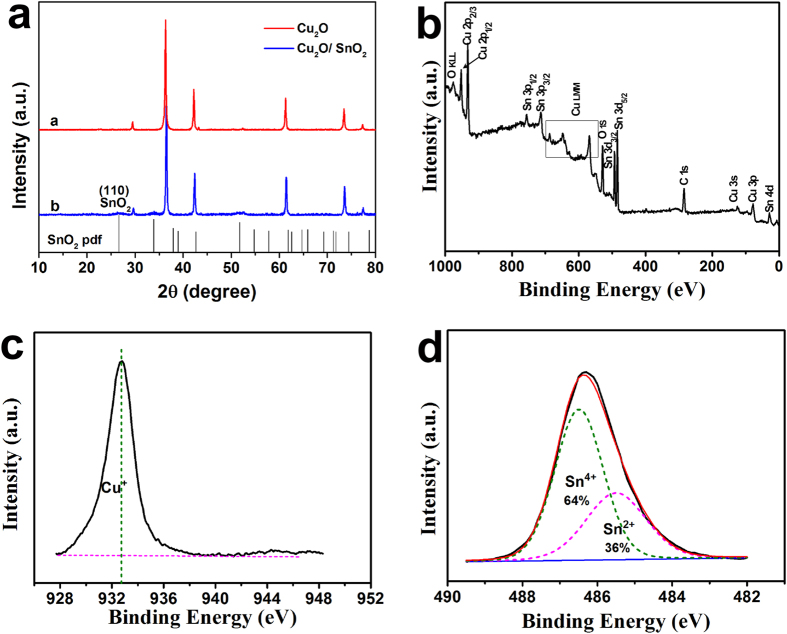
(**a**) X-ray diffraction pattern of as-synthesized Cu_2_O nanocubes, compared with that of SnO_2_ nanoparticle-decorated Cu_2_O nanocubes. (**b**) XPS survey spectrum of the Cu_2_O/SnO_2_ sample. (**c**) and (**d**) High resolution XPS spectrum over Cu 2*p*_3/2_ peak and Sn 3*d*_5/2_ peak, respectively.

Figure 2a shows the X-ray diffraction pattern of as-synthesized Cu_2_O nanocubes, compared with that of SnO_2_ nanoparticle-decorated Cu_2_O nanocubes. For both samples, no diffraction peaks of CuO or Cu could be detected. All diffraction peaks in curve a belonged to the fcc Cu_2_O phase (PDF Card No. 05-0667), and the strong and sharp peaks indicated that these Cu_2_O nanocubes had a high degree of crystallinity. After the decoration with SnO_2_ nanoparticles, several new diffraction peaks emerged in curve b, which could be readily indexed to tetragonal rutile structure of SnO_2_ (PDF Card No. 41-1445). These peaks had relatively weak intensities due to the much smaller size of SnO_2_ nanoparticles, compared with Cu_2_O nanocubes. No other diffraction peak could be observed, which confirmed that the final product was composed of Cu_2_O and SnO_2_. The amount of SnO_2_ nanoparticles in the Cu_2_O/SnO_2_ sample was measured by the sodium diethydlthiocabamate spectrophotometric method, and SnO_2_:Cu_2_O molar ratio was determined at ~0.15: 1.

The chemical composition and element valence states in SnO_2_ nanoparticle-decorated Cu_2_O nanocubes were investigated by X-ray photoelectron spectroscopy (XPS). Figure 2b shows the XPS survey spectrum of the Cu_2_O/SnO_2_ sample, which demonstrated clearly the existence of Sn, O, and Cu in the sample. Due to the widespread presence of carbon in the environment, C *1s* peak could also be observed in the XPS survey spectrum. Figure 2c shows the high resolution XPS spectrum over Cu 2*p*_3/2_ peak. The main peak located at 932.7 eV could be attributed to the Cu^+^ 2*p*_3/2_ orbitals^25^. No obvious shake-up satellite peaks on the higher binding energy side could observed, which confirmed no existence of Cu^2+^on the sample surface^26^,^27^. Figure 2d shows the high-resolution XPS spectrum over Sn 3*d*_5/2_ peak. It could be best fitted by the combination of two peaks centered at 486.5 eV and 485.5 eV, which could be assigned to Sn^4+^3*d*_5/2_ peak and Sn^2+^3*d*_5/2_ peak, respectively^28^,^29^. Thus, a small portion of Sn^4+^on the SnO_2_ nanoparticle surface was reduced to Sn^2+^during the sample synthesis and storage under normal ambient condition. The Sn^2+^percentage was determined to be ~36%, while no SnO could be distinguished either in TEM or XRD analysis results. As a surface characterization technique, XPS could determine the surface composition within a very shallow depth. Thus, the existence of Sn^2+^must be on the very surface of SnO_2_ nanoparticles, while the dominant Sn species in the sample existed as Sn^4+^. It had been well reported in literature that Sn^2+^state could be detected on the surface of SnO_2_ nanoparticles due to the oxygen deficiency at the surface of SnO_2_^28^,^29^.

### Optical properties of SnO_2_ nanoparticle-decorated Cu_2_O nanocubes

**Figure 3:**
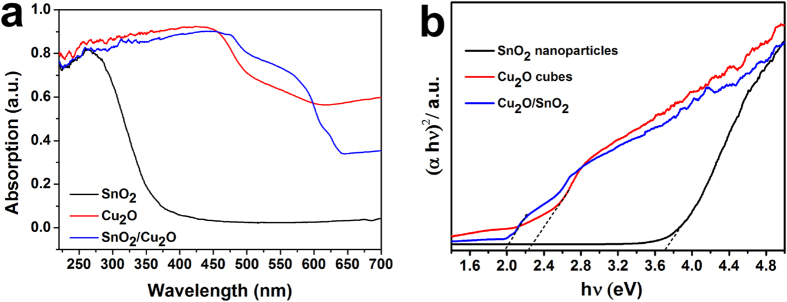
(**a**) The light absorbance (in term of Kubelka-Munk equivalent absorbance units) of the Cu_2_O/SnO_2_ sample, compared with that of the as-synthesized Cu_2_O nanocubes and SnO_2_ nanoparticles. (**b**) Tauc Plots ((*F*(*R*)*hv)^*n*^ vs *hv*) constructed from Fig. 3a.

The optical properties of SnO_2_ nanoparticle-decorated Cu_2_O nanocubes were investigated by measuring their diffuse reflectance spectrum. From the reflectance data, optical absorbance could be approximated by the Kubelka-Munk function, as given by Eq. (4):


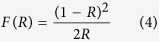


where *R* is the diffuse reflectance^30^. Figure 3a shows the light absorbance (in term of Kubelka-Munk equivalent absorbance units) of the Cu_2_O/SnO_2_ sample, compared with that of the as-synthesized Cu_2_O nanocubes and SnO_2_ nanoparticles. SnO_2_ nanoparticles demonstrated the characteristic spectrum with the fundamental absorbance stopping edge at ~350 nm, so most of their adsorption was within the UV light region^23^. Cu_2_O nanocubes, however, demonstrated a largely enhanced light absorption in the visible light region. Their absorbance stopping edge was found at ~600 nm, which was in accordance with the reported band gap of Cu_2_O at ~2.1 eV^31^. Light absorbance shoulder peaks and tail in the red and near IR regions were observed on the light absorbance spectrum of Cu_2_O nanocubes, which could be attributed to their light scattering from their cubic morphology^32^,^33^. After being decorated with SnO_2_ nanoparticles, the light absorbance behavior of the Cu_2_O/SnO_2_ sample maintained most characteristics of Cu_2_O nanocubes because Cu_2_O was its major component, while the interface charge transfer (IFCT) from SnO_2_ VB to Cu_2_O led to the occurrence of other shoulder peaks^34^. This observation further confirmed the formation of heterojunctions between Cu_2_O and SnO_2_ in this sample. The band gap values of these three photocatalyst samples were determined by the construction of Tauc Plots ((*F*(*R*)*hv)^*n*^ vs *hv*) from their light absorbance data^35^. Figure 3b shows Tauc Plots of these three photocatalyst samples, respectively. As direct band gap semiconductors, *n* should be taken as 2 for both Cu_2_O and SnO_2_^31^,^35^. Thus, the extrapolation of the linear region to the photon energy axis could yield their bandgap values of ~3.71 eV for SnO_2_ nanoparticles, ~2.27 eV for Cu_2_O nanocubes and ~2.16 eV for the Cu_2_O/SnO_2_ sample, consistent with their light absorption performances.

### Photocatalytic disinfection of *Staphylococcus aureus* bacteria under visible light illumination

**Figure 4:**
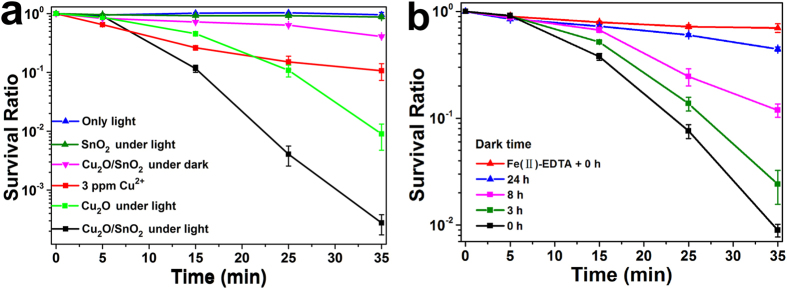
(**a**) The survival ratio of S. aureus cells with the treatment by the Cu_2_O/SnO_2_ sample under visible light illumination, compared with that without photocatalyst under visible light illumination, that by the Cu2O/SnO_2_ sample in the dark, that by SnO_2_ nanoparticles under visible light illumination, that by Cu_2_O nanocubes under visible light illumination, and that by Cu^2+^ ion with 3 ppm concentration. (**b**) The *S. aureus* cell survival ratios in the dark treated by pre-illuminated Cu_2_O/SnO_2_ samples after being stored in the dark for various times, compared with that with a H_2_O_2_ scavenger, EDTA-Fe(II) (0.1 M), in the *S. aureus* cell suspension treated by the pre-illuminated Cu_2_O/SnO_2_ sample in the dark with the dark storage time of 0 h.

The superior photocatalytic performance of SnO_2_ nanoparticle-decorated Cu_2_O nanocubes was demonstrated by their photocatalytic disinfection effect on the viability of *S. aureus* cells, which is a common pathogenic coccus that could cause nonspecific infection and nosocomial infection^13^,^36^. The photocatalytic disinfection was conducted by exposing *S. aureus* cells suspended in 0.9% NaCl solution with the photocatalyst under visible light illumination for varying time intervals. The survival ratio of *S. aureus* was determined by the ratio of *N*_t_/*N*_0_, where *N*_0_ and *N*_t_ were the numbers of colony-forming units at the initial and each following time interval, respectively. Figure 4a shows the survival ratio of *S. aureus* cells under different treatment conditions. When no photocatalyst was present, no obvious change was observed for the survival ratio of *S. aureus* cells under visible light illumination, which suggested that visible light itself could not disinfect *S. aureus* cells. SnO_2_ nanoparticles also did not show an obvious bactericidal effect under visible light illumination because they could not be activated by visible light due to their wide band gap of ~3.71 eV. Cu_2_O nanocubes demonstrated an obvious bactericidal effect under visible light illumination. After 35 min treatment, the survival ratio of *S. aureus* cells dropped to ~10^−2^, which could be mainly attributed to their strong visible light absorption (band gap of ~2.27) and subsequent photocatalytic activity under visible light illumination. For the Cu_2_O/SnO_2_ sample, it only showed a moderate bactericidal effect on *S. aureus* cells without light illumination. After 35 min treatment, the survival ratio of *S. aureus* cells was still ~40.8%, which should come from the well-known bactericidal effect of copper-based oxides. The detailed discussion of Cu ion leakage from the Cu_2_O/SnO_2_ sample during the treatment process and its minor contribution to the disinfection of *S. aureus* cells can be found in the supplementary information. Under visible light illumination, however, the Cu_2_O/SnO_2_ sample demonstrated a much faster bactericidal effect on *S. aureus* cells. After a relatively slow dropping for the first 5 min of the treatment, the survival ratio of *S. aureus* cells dropped sharply and continuously with the increase of the treatment time. The survival ratio of *S. aureus* cells dropped to ~2.78*10^−4^ after only 35 min treatment, more than 3 magnitudes lower than that without visible light illumination. From the comparison, it is clear that the demonstrated superior bactericidal effect of SnO_2_ nanoparticle-decorated Cu_2_O nanocubes on *S. aureus* cells under visible light illumination could be mainly attributed to their superior photocatalytic disinfection performance, not the modest bactericidal effect from the Cu_2_O nanocubes itself in this composite photocatalyst system. The formation of *p*-*n* heterojunctions Cu_2_O and SnO_2_ could largely enhance the charge carrier separation, which resulted in the largely enhanced photocatalytic *S. aureus* cell disinfection performance of the Cu_2_O/SnO_2_ sample, compared with pure Cu_2_O nanocubes. To further demonstrate the superior photocatalytic performance of the Cu_2_O/SnO_2_ sample, its photocatalytic degradation effect on an antibiotic sulfamethoxazole (SMX) was also examined under visible light illumination, and the results can be found in the supplementary information.

### Post-illumination photocatalytic “memory” disinfection of *Staphylococcus aureus* bacteria in the dark

As expected, the post-illumination photocatalytic “memory” disinfection of *S. aureus* cells in the dark was observed for SnO_2_ nanoparticle-decorated Cu_2_O nanocubes. In this experiment series, Cu_2_O/SnO_2_ samples were firstly illuminated by the same visible light source used in the photocatalytic disinfection experiment for ~3 h. Then, the visible light was shut off and the samples were stored in a dark environment for 0 h, 3 h, 8 h, and 24 h, respectively, before they were used to conduct disinfection experiments on fresh *S. aureus* cells in the dark under the same experimental setup as the photocatalytic disinfection experiment only without the light illumination. Figure 4b shows the *S. aureus* cell survival ratios in the dark treated by pre-illuminated Cu_2_O/SnO_2_ samples after being stored in the dark for various times. It demonstrated clearly that pre-illuminated Cu_2_O/SnO_2_ samples could effectively disinfect *S. aureus* cells in the dark even after the visible illumination was shut off. When the dark storage time was 0 h, the survival ratio of *S. aureus* cells dropped to ~0.9% after only 35 min treatment in dark. With the increase of the dark storage time from 3 h, 8 h, to 24 h, the bactericidal effect of the pre-illuminated Cu_2_O/SnO_2_ sample in the dark dropped gradually, and the survival ratio of *S. aureus* cells after 35 min treatment in dark increased from ~2.4%, ~11.9%, to ~44.5%, respectively. In the dark, the survival ratio curve of *S. aureus* cells treated by the pre-illuminated Cu_2_O/SnO_2_ sample with the dark storage time of 24 h was close to that treated by the same photocatalyst without pre-illumination. This observation clearly demonstrated that the post-illumination disinfection capability of the Cu_2_O/SnO_2_ sample in the dark relied on its “memory” of the visible light illumination prior to the dark environment, not the photocatalytic material itself. The observed post-illumination photocatalytic “memory” effect in the dark of SnO_2_ nanoparticle-decorated Cu_2_O nanocubes with pre-illumination was stronger than that of TiON/PdO and Cu_2_O-NS/TiO_2_-NI photocatalysts developed in our previous work^14^,^15^,^16^. In these photocatalytic material systems, electron trapping and release occurred when the visible light illumination was on and off, respectively, which enhanced their photocatalytic performances under visible light illumination and resulted in their post-illumination photocatalytic “memory” in the dark.

### The production and role of H_2_O_2_ in the photocatalytic disinfection under visible light illumination and post-illumination photocatalytic “memory” disinfection in the dark of *Staphylococcus aureus* bacteria

**Figure 5:**
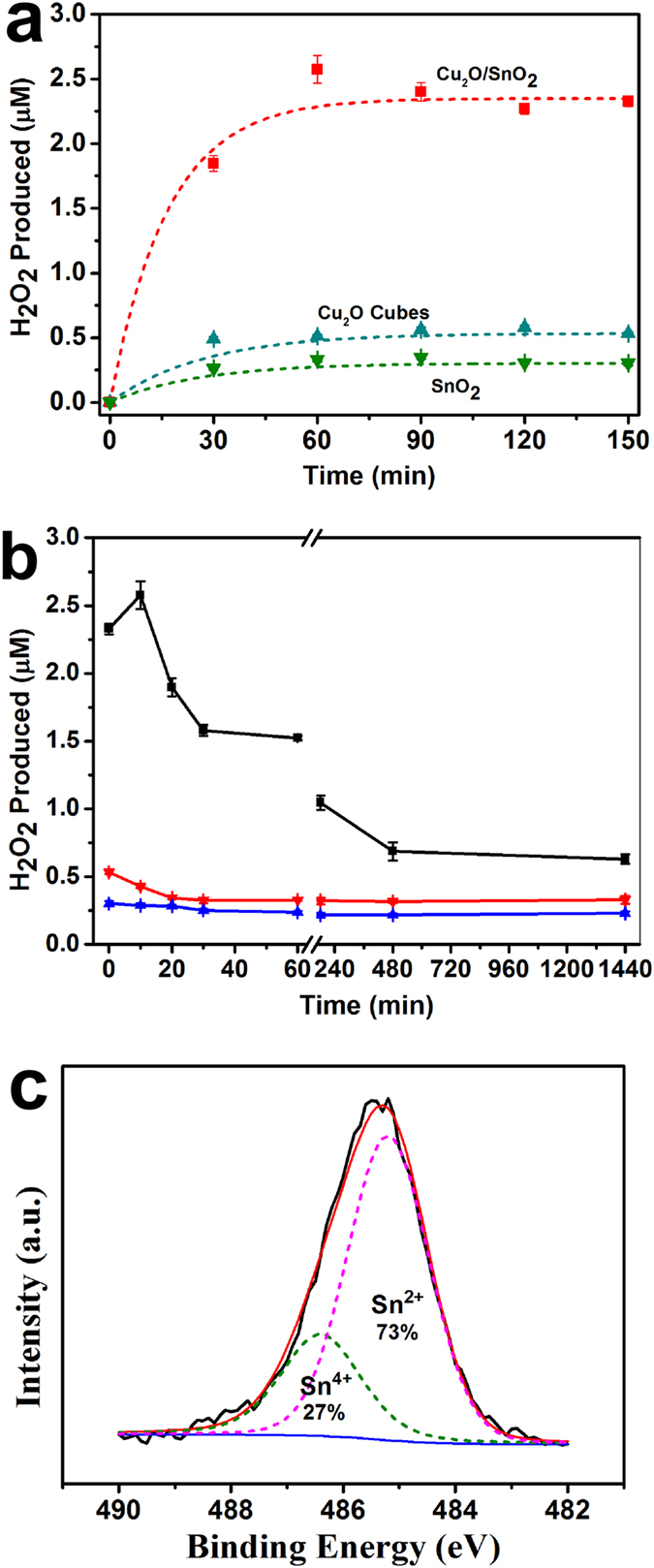
The H_2_O_2_ concentrations in the test solution by the Cu_2_O/SnO_2_ sample, the as-synthesized Cu_2_O nanocubes, and SnO_2_ nanoparticles, respectively: (**a**) under visible light illumination, and (**b**) in the dark for up to 24 h after being illuminated under visible light for 3 h. (**c**) The high resolution XPS scan over Sn *3d* peaks under visible light illumination.

Generally, various reactive oxygen species, such as H_2_O_2_, ^•^O_2_^−^, ^•^OH, h^+^, and e^−^, are produced *in situ* during the photocatalytic process^37^. To verify the occurrence of the two-electron reduction of O_2_ to H_2_O_2_ by the Cu_2_O/SnO_2_ sample, the concentrations of H_2_O_2_ in the test solution were examined by a colorimetric DPD method under visible light illumination and in the dark, respectively. Figure 5a shows the H_2_O_2_ concentrations in the test solution under visible light illumination by the Cu_2_O/SnO_2_ sample, the as-synthesized Cu_2_O nanocubes, and SnO_2_ nanoparticles, respectively. It demonstrated that the H_2_O_2_ yield increased at first and then became relatively stable with prolonged illumination time because the consumption of H_2_O_2_ was in parallel with its production, which gradually reached the equilibrium. The H_2_O_2_ production by the Cu_2_O/SnO_2_ sample was much higher than Cu_2_O nanocubes and SnO_2_ nanoparticles. The measured equilibrium H_2_O_2_ concentration was ~2.33 μM for the Cu_2_O/SnO_2_ sample, ~772% as that of SnO_2_ nanoparticles and ~438% as that of the Cu_2_O nanocubes. The observed enhancement of H_2_O_2_ production could be attributed to the transfer of photogenerated electrons from Cu_2_O to SnO_2_ under visible light illumination due to the formation of Cu_2_O/SnO_2_ *p*-*n* heterojunction, which in turn could enhance photogenerated electron-hole pair separation in Cu_2_O and increase the production of ^•^OH because more holes on the valence band of Cu_2_O could have the chance to migrate to the surface of Cu_2_O and react with H_2_O to form ^•^OH. Thus, the Cu_2_O/SnO_2_ sample demonstrated much better photocatalytic performances on the degradation of SMX and disinfection of *S. aureus* cells than Cu_2_O nanocubes under visible light illumination. It must be pointed out that the total amount of H_2_O_2_ produced should be much higher than the measured equilibrium concentration value because it was consumed *in situ* in the system with its generation.

Figure 5b shows the H_2_O_2_ concentrations in the test solution in the dark for an extended period of time up to 24 h after the visible light illumination was turned off by the Cu_2_O/SnO_2_ sample, the as-synthesized Cu_2_O nanocubes, and SnO_2_ nanoparticles, respectively. For the as-synthesized Cu_2_O nanocubes and SnO_2_ nanoparticles, their production of H_2_O_2_ was limited and the H_2_O_2_ concentrations dropped quickly within the first 30 min in the dark. This observation was similar to the previous reports on Se^17^ and Bi^18^, in which very limited charge carriers could remain at their surfaces, produce ROSs after the illumination was shut off, and be consumed quickly. After 30 min, the equilibrium H_2_O_2_ concentrations was ~0.33 μM for Cu_2_O nanocubes and ~0.25 μM for SnO_2_ nanoparticles for the whole experiment time up to 24 h in the dark, which may reflect the H_2_O_2_ concentration in the environment background. For the Cu_2_O/SnO_2_ sample, however, a completely different H_2_O_2_ production behavior was observed. The H_2_O_2_ concentration increased quickly for the first 10 min in the dark, which could be attributed to the quick release of trapped electrons from SnO_2_ to react with O_2_. Then, it decreased gradually afterwards up to 24 h in the dark as long as the experiment explored. Even after 24 h in the dark, the H_2_O_2_ concentration still reached ~0.63 μM, much higher than the H_2_O_2_ concentration in the environment background. Thus, the Cu_2_O/SnO_2_ sample could still be active to disinfect *S. aureus* cells in the dark even after the light illumination was off for 24 h as shown in Fig. 4b. The gradual decrease of the H_2_O_2_ concentration with the increase of dark time also clearly demonstrated that the post-illumination disinfection capability of the Cu_2_O/SnO_2_ sample in the dark relied on its “memory” of the visible light illumination prior to the dark environment, which was the trapping of photogenerated electrons.

Figure 5c shows the high resolution XPS scan over Sn *3d* peaks under visible light illumination. The Sn^4+^/Sn^2+^ratio was determined to be ~27:73. Compared to that without illumination as shown in Fig. 2d, a large part of Sn^4+^was reduced to Sn^2+^, which came from the transfer of photogenerated electrons from Cu_2_O to SnO_2_ under visible light illumination and the subsequent trapping of part of these electrons by SnO_2_. After the light illumination was shut off, these trapped electrons could be gradually released and react with O_2_ to produce H_2_O_2_ as shown in Fig. 5b. To further confirm the production of H_2_O_2_ and its major contribution to the disinfection of *S. aureus* cells by the Cu_2_O/SnO_2_ sample in the dark after the illumination was shut off, a H_2_O_2_ scavenger, EDTA-Fe(II), was used to examine if its existence could affect the survival ratio of *S. aureus* cells^38^. 0.1 mM EDTA-Fe(II) was added into the *S. aureus* cell suspension, and the pre-illuminated Cu_2_O/SnO_2_ sample was used to conduct the disinfection experiment in the dark. As shown in Fig. 5b, the presence of EDTA-Fe(II) largely enhanced the survival ratio of *S. aureus* cells, which was very close to that treated by the Cu_2_O/SnO_2_ sample without pre-illumination in the dark. This observation further confirmed that H_2_O_2_ was the dominant ROS involved in the photocatalytic “memory” disinfection of *S. aureus* cells by the pre-illuminated Cu_2_O/SnO_2_ sample in the dark.

## Discussion

**Figure 6:**
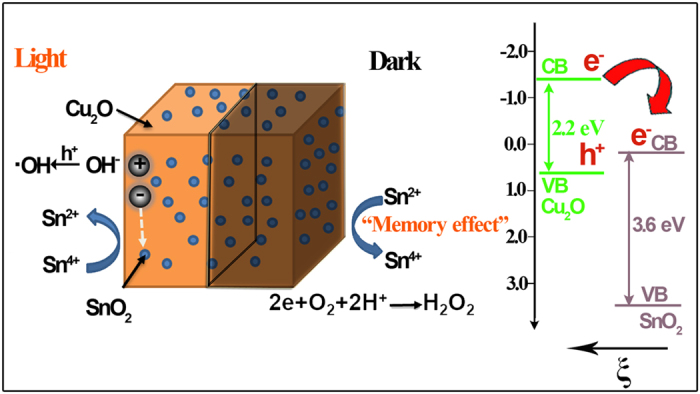
The proposed energy band structure of the Cu_2_O/SnO_2_ *p*-*n* heterojunction, the photocatalytic activity enhancement mechanism under visible light illumination, and the post-illumination photocatalytic “memory” mechanism in the dark.

Figure 6 shows the proposed energy band structure of the Cu_2_O/SnO_2_ *p*-*n* heterojunction, the photocatalytic activity enhancement mechanism under visible light illumination^39^,^40^, and the post-illumination photocatalytic “memory” mechanism in the dark. When *p*-type Cu_2_O and *n*-type SnO_2_ formed a heterojunction, charge carrier concentration gradient occurred at the interface. Thus, the diffusion of electrons from SnO_2_ to Cu_2_O and the diffusion of holes with the opposite direction happened until reaching the equilibrium, and an inner electric field (ξ) was built at the interface as demonstrated in Fig. 6. Under visible light illumination, only Cu_2_O was excited to produce electron-hole pairs. The combined effect from both the large conduction band potential difference (~1.5 eV) and the inner electric field (ξ) provided a strong driving force for photogenerated electrons to transfer from the conduction band of Cu_2_O to that of SnO_2_ and be trapped there as verified by experimental evidences of the H_2_O_2_ production and XPS analysis on Sn chemical status change from Sn^4+^to Sn^2+^. Thus, the photogenerated electron-hole pairs were separated effectively, and a largely enhanced photocatalytic performance was observed on the Cu_2_O/SnO_2_ sample for its degradation of SMX and disinfection of *S. aureus* cells, compared with pure Cu_2_O nanocubes, which was very similar to our previous report on Cu_2_O-NS/TiO_2_-NI photocatalyst system^16^. When the visible light illumination was shut off, the trapped electrons could be released from SnO_2_ and the two-electron reduction of O_2_ could happen by its reaction with these released electrons due to their matched reduction potentials, which was verified by the continuous production of H_2_O_2_ in the dark for more than 24 h. So the Cu_2_O/SnO_2_ sample could demonstrate the post-illumination photocatalytic “memory” disinfection of *S. aureus* cells in the dark after the illumination was shut off.

The ROS production in the dark by the Cu_2_O/SnO_2_ sample developed in this study was quite different with that of previous reported photocatalysts with the post-illumination photocatalytic “memory” effect^14^,^15^,^16^,^19^. For TiON/PdO^14^,^15^, Cu_2_O-NS/TiO_2_-NI^16^, and I-TiO_2_^19^, photogenerated electrons were released from the decoration components in the dark and one-electron reduction of O_2_ happened to produce ^•^O_2_^−^ and subsequently ^•^OH as ROSs, which required the decoration components had the conduction band potential negative than the one-electron reduction potential of O_2_. For the Cu_2_O/SnO_2_ sample, however, photogenerated electrons were released from SnO_2_ in the dark and two-electron reduction of O_2_ happened to produce H_2_O_2_ as ROS. Thus, this work demonstrated that a decoration component with the conduction band potential less positive than the two-electron reduction potential of O_2_ could also be effective to generate the post-illumination photocatalytic “memory” effect from the production of H_2_O_2_ in the dark.

This finding suggested that the selection of potential decoration components to construct photocatalyst systems with the post-illumination photocatalytic “memory” effect could be largely expanded to more semiconductors, such as SnO_2_, WO_3_, CuWO_4_, BiWO_6_, CeO_2_, etc. Although they do not have conduction band potentials negative than the one-electron reduction potential of O_2_, so trapped electrons released by them could not reduce O_2_ in the dark to produce ^•^O_2_^−^ and subsequently ^•^OH. However, electrons trapped on them could be released and then reduce O_2_ in the dark to produce the reactive oxygen species of H_2_O_2_ because their conduction band potentials are less positive than the two-electron reduction potential of O_2_. Thus, novel photocatalyst systems with the post-illumination photocatalytic “memory” effect could be designed based on these decoration components paired with light absorber components of proper conduction band potentials in which photogenerated electrons could transfer from the light absorber component to the decoration component for subsequent trapping under light illumination and release after the illumination was shut off. Furthermore, different photocatalyst systems with the post-illumination photocatalytic “memory” effect could be designed by modulating the conduction band potential of the decoration component to produce different kinds of ROSs for the optimized performance for various applications.

In summary, a novel composite photocatalyst composed of Cu_2_O nanocubes decorated with SnO_2_ nanoparticles was successfully created, in which Cu_2_O served as the main visible light absorber, while SnO_2_ nanoparticle decoration formed *p*-*n* heterojunction of good contact with Cu_2_O nanocubes. The combined effect from both their large conduction band potential difference and the inner electric field provided a strong driving force for photogenerated electrons to transfer from the conduction band of Cu_2_O to that of SnO_2_ and be trapped there under visible light illumination. Thus, a largely enhanced photocatalytic performance was observed on these Cu_2_O/SnO_2_ photocatalysts as demonstrated by its disinfection of *S. aureus* cells and degradation of SMX, compared with pure Cu_2_O nanocubes. When the visible light illumination was turned off, trapped electrons could be released from SnO_2_ and react with O_2_ to produce H_2_O_2_ in the dark for more than 24 h, and the Cu_2_O/SnO_2_ sample demonstrated a strong post-illumination photocatalytic “memory” disinfection of *S. aureus* cells in the dark. This work demonstrated that the selection of potential decoration components to construct photocatalyst systems with the post-illumination photocatalytic “memory” effect could be largely expanded to semiconductors with conduction band potentials less positive than the two-electron reduction potential of O_2_. With high efficiency and relatively low cost/energy consumption, photocatalysts with the post-illumination photocatalytic “memory” effect could have the potential for a broad range of environmental applications which require the continuous activity in the dark for an extended period of time.

## Methods

### Chemicals and materials

Copper(II) chloride dihydrate (CuCl_2_•2H_2_O, 99%, Sinopharm Chemical Reagent Co., Ltd., Shanghai, P. R. China) was used as the Cu source, polyvinylpyrrolidon (PVP k30, Sinopharm Chemical Reagent Co., Ltd., Shanghai, P. R. China) was used as the surfactant, sodium hydroxide (NaOH, 96%, Sinopharm Chemical Reagent Co., Ltd., Shanghai, P. R. China) was used as the precipitation reagent, and L-ascorbic acid (99.7%, Aladdin Industrial Corporation Co. Ltd., Shanghai, P. R. China) was used as the reducing agent in the synthesis of Cu_2_O nanocubes, respectively. Deionized (DI) water was used as the solvent in this process. Potassium stannate trihydrate (K_2_SnO_3_·3H_2_O, 99.5%, Aladdin Industrial Corporation Co. Ltd., Shanghai, P. R. China) was used as the Sn source and Ethyl acetate (C_4_H_8_O_2_, 99.5%, Sinopharm Chemical Reagent Co., Ltd., Shanghai, P. R. China) was used as the hydrolytic reagent in the formation of SnO_2_ nanoparticles. Sulfamethoxazole (SMX, 98%, Aladdin Industrial Corporation Co. Ltd., Shanghai, P. R. China) was used as the target organic pollution compound for the investigation of the sample’s visible light-induced photocatalytic activity. Sodium diethydlthiocabamate (DDTC, 99%, Sinopharm Chemical Reagent Co., Ltd., Shanghai, P. R. China) was used to detect the Cu^2+^concentration. Horseradish peroxide (POD,>200 units/mg, Aladdin Industrial Corporation Co. Ltd., Shanghai, P. R. China) and N,N-diethyl-p-phenylenediamine sulfate (DPD, 98%, Aladdin Industrial Corporation Co. Ltd., Shanghai, P. R. China) were used as the chromogenic reagents, while phenol (99%, Alfa Aesar Chemical Ltd., Tianjin, P. R. China) was used as the hole scavenger in the detection of the H_2_O_2_ concentration experiment.

### Synthesis of Cu_2_O nanocubes

Cu_2_O nanocubes were synthesized by a modified process based on a previous report^41^. In a typical experiment, 0.3 g PVP was first dissolved in 270 mL DI water, and 30 mL of 0.02 M CuCl_2_•2H_2_O solution was added into the PVP solution. Then, 3.6 mL of 0.6 M NaOH was added drop wise (1 drop/s) into the above mixture solution with continual stirring. Finally, 4 mL of 0.3 M L-ascorbic acid was added drop wise into the mixture solution and it was further stirred for 5 min before being centrifuged at 9,500 rpm for 5 min. All of these procedures were carried out in a water bath at 35 °C. The obtained yellow precipitates were washed with excessive DI water and ethanol for several times to remove unreacted chemicals and PVP surfactants.

### Preparation of SnO_2_ nanoparticle-decorated Cu_2_O nanocubes

The obtained Cu_2_O nanocubes were dispersed in a mixture solvent consisting of 15 mL of DI water and 10 mL of absolute ethanol with the aid of ultrasonication for 15 min. Then, 2.5 mL of 0.01 M potassium stannate trihydrate (K_2_SnO_3_·3H_2_O) solution was slowly dropped into the Cu_2_O suspension and stirred for 10 min. After thorough mixing, 0.2 mL of ethyl acetate (C_4_H_8_O_2_) was added drop wise into the mixture under vigorous stirring for 1 h. Finally, the suspension was transferred into a 50 mL Teflon-lined stainless steel autoclave, and heated at 170 ^o^C for 6 h in an oven. After the reaction, the products were collected, went through several rinse-centrifugation cycles with DI water and ethanol separately, and then dried at 40 ^o^C for 12 h in a vacuum oven. Bare SnO_2_ nanoparticles were also prepared via the same hydrothermal process without the adding of Cu_2_O nanocubes and were used as a reference material for the photocatalytic testing.

### Materials characterization

The crystal structures of the as-prepared samples were analyzed by X-ray diffraction (XRD) on a D/MAX-2004 X-ray powder diffractometer (Rigaku Corporation, Tokyo, Japan) with Ni-filtered Cu Kα (λ = 1.54178 Å) radiation at 56 kV and 182 mA. The morphologies of the as-prepared samples were observed by the transmission electron microscopy (TEM). TEM observations were conducted on a JEOL 2100 TEM (JEOL Ltd., Tokyo, Japan) operated at 200 kV with point-to-point resolution of 0.28 nm, and TEM samples were prepared by dispersing a thin film of these powder samples on Ni grids. A Tecnai G2 F20 transmission electron microscope (FEI, Acht, The Netherlands) was used to obtain high-resolution TEM (HRTEM) images of samples. X-ray photoelectron spectroscopy (XPS) measurements were conducted using an ESCALAB 250 X-ray photoelectron spectrometer (Thermo Fisher Scientific Inc., Waltham, MA, U. S. A.) with an Al Kα anode (1486.6 eV photon energy, 300 W). The UV-vis spectra of samples and concentration of SMX were measured on a UV-2550 spectrophotometer (Shimadzu Corporation, Kyoto, Japan).

### Photocatalytic disinfection of Staphylococcus aureus (*S. aureus*) bacteria under visible light illumination

Wild-type *S. aureus* (CMCC(B)26003, China national standard material network, P. R. China) were used for photocatalytic disinfection experiments. After overnight culture, cells were diluted to a cell suspension (ca.10^7^ cfu/mL) in 0.9% NaCl solution prior to the use for photocatalytic disinfection experiments. All solid or liquid materials had been autoclaved for 30 min at 121 ^o^C before use. The same visible light source was used as in the photocatalytic degradation of SMX. In the photocatalytic disinfection of *S. aureus* bacteria experiment, aliquot of 10 mL *S. aureus* cell suspension was pipetted onto a sterile 50 × 10 mm petri dish with the photocatalytst sample, which was first spin coated at the bottom of the dish. A fixed concentration of ~0.2 mg photocatalyst/mL *S. aureus* solution was used in this experiment. At regular time intervals, 100 μL of aliquots of the powder-treated cell suspensions were withdrawn in sequence. After appropriate dilutions in 0.9% NaCl solution, aliquot of 100 μL was spread onto an agar medium plate and incubated at 37 ^o^C for 15 h. The number of viable cells in terms of colony-forming units was counted. Tests were also performed in the dark in the presence of the photocatalyst for comparison. Analyses were in triplicate, and control runs were carried out each time under the same experiment conditions, but without any photocatalytic materials.

### Photocatalytic “memory” disinfection of Staphylococcus aureus (*S. aureus*) bacteria in the dark

For *S. aureus* bacteria disinfection under dark environment, the Cu_2_O/SnO_2_ sample was firstly illuminated by the same lamp for ~3 h. Then, the lamp was shut off and they were used to conduct disinfection experiments in the dark over fresh *S. aureus* cell suspensions (ca. 10^7^ cfu/mL) either immediately or after being kept in dark for 3, 8 and 24 h. In some experiments, Fe(II)-EDTA (0.1 mM) was added for the removal of H_2_O_2_ to examine the reactive oxygen species^37^,^38^. All experimental conditions were the same as that for the photocatalytic disinfection of *S. aureus* bacteria, but without the visible light illumination.

### Detection of the hydrogen peroxide (H_2_O_2_) concentration

The colorimetric DPD method based on the horseradish peroxidase (POD) catalyzed oxidation of *N*,*N*-diethyl-*p*-phenylenediamine (DPD) was used for the detection of *in situ* photogenerated H_2_O_2_^42^. 0.1 g *N*,*N*-diethyl-*p*-phenylenediammonium sulfate was first dissolved in 10 mL of 0.1 M H_2_SO_4_ solution, and 10 mg POD was dissolved in 10 mL DI water. Both DPD and POD solutions were stored in the dark at 4 °C in a refrigerator and replaced with fresh solutions at weekly intervals. For the detection of H_2_O_2_ concentration, 5 mL aliquot of the test solution was pipetted into a 10 mL test tube, and mixed with 0.5 mL phosphate buffer solution (0.5 M KH_2_PO_4_ and 0.5 M K_2_HPO_4_) to yield a pH of ~6.0. 50 μL DPD solution was then added into the mixture solution, followed by the addition of 50 μL POD solution with shaking for 10 sec. The solution was then settled for 30 sec before the UV-vis spectrum measurement. The H_2_O_2_ concentration could be quantified by the UV-2550 spectrophotometer monitoring the absorption maximum at *λ*_max_ of 551 nm.

## Additional Information

**How to cite this article**: Liu, L. *et al.* Post-illumination activity of SnO_2_ nanoparticle-decorated Cu_2_O nanocubes by H_2_O_2_ production in dark from photocatalytic “memory”. *Sci. Rep.* **6**, 20878; doi: 10.1038/srep20878 (2016).

## Supplementary Material

Supplementary Information
