# Supplementary material for: Post-illumination activity of SnO2 nanoparticle-decorated Cu2O nanocubes by H2O2 production in dark from photocatalytic “memory”
Source: Sci Rep. 2016 Feb 16;6:20878. doi: 10.1038/srep20878 (PMC4754728; doi:10.1038/srep20878)
Supplement: Supplementary Information [file srep20878-s1.doc]

**Post-illumination activity of SnO_2_ nanoparticle-decorated Cu_2_O nanocubes by H_2_O_2_ production in dark from photocatalytic “memory**”

Lingmei Liu^1^, Wuzhu Sun^1^, Weiyi Yang^1^, Qi Li^1,*^ & Jian Ku Shang^2^

^1^*Environment Functional Materials Division*

*Shenyang National Laboratory for Materials Science*

*Institute of Metal Research, Chinese Academy of Sciences, Shenyang 110016, P. R. China*

^2^*Department of Materials Science and Engineering*

*University of Illinois at Urbana-Champaign, Urbana, Illinois 61801, USA*

^*^Corresponding author: E-mail address: [qili@imr.ac.cn](mailto:qili@imr.ac.cn) (Q. Li)

Phone: +86-24-83978028, Fax: +86-24-23971215.

Postal address: 72 Wenhua Road, Shenyang, Liaoning Province, 110016, P. R. China.

**Results**

**Photocatalytic degradation of sulfamethoxazole (SMX) under visible light illumination.** As an important prescribed antibiotic used worldwide to treat bronchitis and urinary tract infections, sulfamethoxazole (SMX) has been widely used as veterinary medicine for prevention and treatment of infections or as a growth promoter^S1,S2^. However, SMX has been identified in surface water at concentrations ranging from 0.01 to 2.0 μg/L in different countries because of its low biodegradability, so it could be accumulated in various organisms and cause environmental pollution and human diseases^S3^. The photocatalytic activity of SnO_2_ nanoparticle-decorated Cu_2_O nanocubes was further demonstrated by their degradation effect on SMX under visible light illumination (*λ *> 400 nm). Fig. S1a shows the adsorption spectrum of SMX solution treated by the Cu_2_O/SnO_2_ sample under visible light illumination. Before the illumination was on, the Cu_2_O/SnO_2_ sample was mixed with the SMX solution in the dark for an hour to establish the adsorption-desorption equilibrium. It showed that the characteristic absorption peak of SMX at ~ 257 nm decreased gradually with the increase of the illumination time, which clearly demonstrated that SMX was photocatalytically degraded by the Cu_2_O/SnO_2_ sample under visible light illumination.

Fig. S1b summaries the residue SMX concentration vs. treatment time under different treatment conditions. When there was no photocatalyst presence, SMX solution kept most of its initial concentration under visible light illumination. The SMX adsorption onto these photocatalysts was examined, which demonstrated that the SMX adsorption-desorption equilibrium could be established within an hour and the SMX concentration decrease from its adsorption on these photocatalysts was not over 5%. Under visible light illumination, SnO_2_ nanoparticles displayed a weak SMX removal capability. After 180 min treatment, the residual SMX concentration was still ~ 95%. Cu_2_O nanocubes demonstrated a better photocatalytic degradation effect on SMX under visible light illumination. After 180 min treatment, the residual SMX concentration dropped to ~ 80%, which could be attributed to their strong visible light absorption and subsequent photocatalytic activity under visible light illumination. The Cu_2_O/SnO_2_ sample demonstrated the best photocatalytic degradation effect on SMX under visible light illumination. After 180 min treatment, the residual SMX concentration dropped to ~ 50%, which could be attributed to the enhanced charge carrier separation by the formation of Cu_2_O/SnO_2_ *p*-*n* heterojuctions^S4,S5,S6^.

The slope of the SMX degradation curve in Fig. S1b represents the SMX degradation rate at certain treatment time. The photocatalytic activity enhancement could be further demonstrated quantitatively by the initial SMX degradation rates for different photocatalysts with similar initial SMX concentrations. When SnO_2_ nanoparticles were used, the initial SMX degradation rate was ~ 0.026 mg/(g∙min). When Cu_2_O nanoparticles were used, the initial SMX degradation rate increased to ~ 0.062 mg/(g∙min), representing a ~ 238% increase as that of SnO_2_ nanoparticles. The initial SMX degradation rate by the Cu_2_O/SnO_2_ sample further increased to ~ 0.097 mg/(g∙min), ~ 373% as that of SnO_2_ nanoparticles and ~ 156% as that of the bare Cu_2_O nanocubes. The result clearly demonstrated the enhancement of photocatalytic capability of the Cu_2_O/SnO_2_ sample by the decoration of SnO_2_ nanoparticles on Cu_2_O nanocubes.


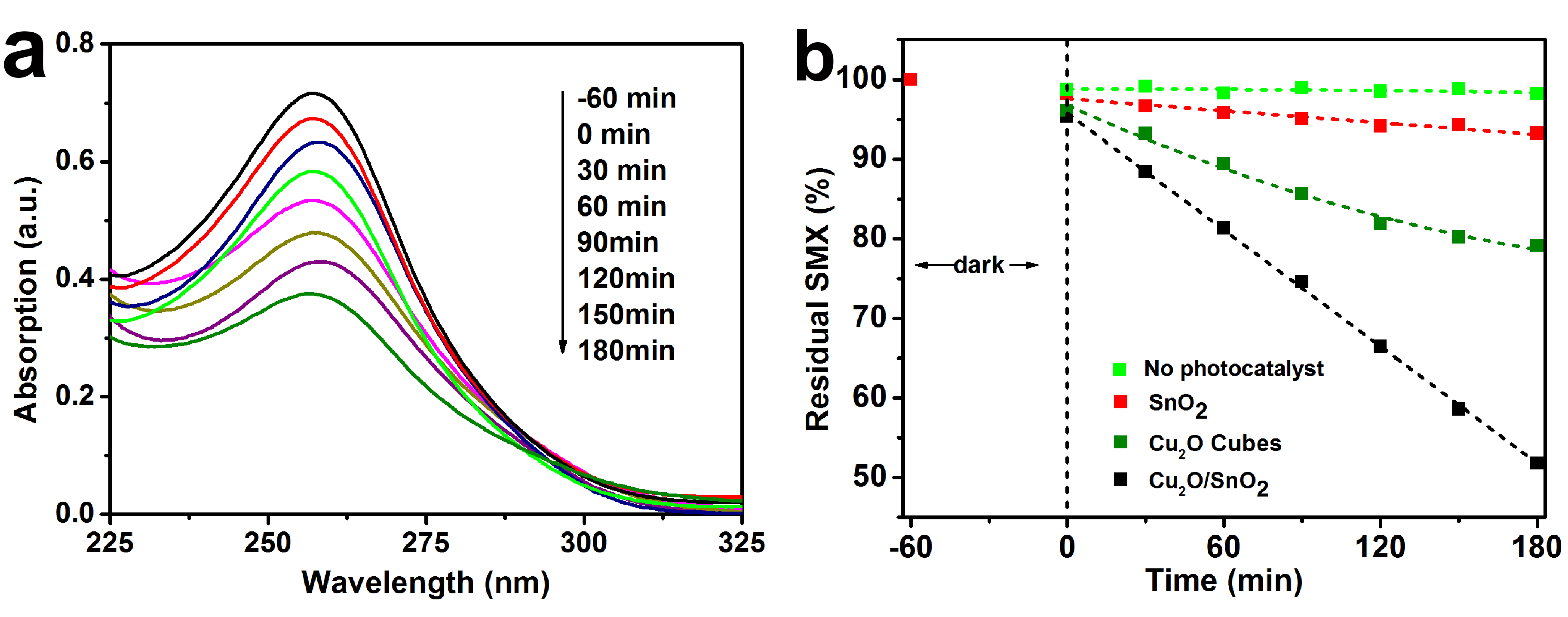


**Figure S1.** (a) The adsorption spectra of SMX solution treated by the Cu_2_O/SnO_2_ sample under visible light illumination for different treatment time. (b) The residue SMX concentration vs. treatment time under different treatment conditions.

**The Influence of Cu^2+^ in Photocatalytic Disinfection of *Staphylococcus aureus* Bacteria.** It is well known that copper ions at high concentrations are toxic to microorganisms because it could bind to biomolecules, such as proteins and nucleic acids, and affect their bio-functions^S7^. Thus, the possible leakage of copper ions into the cell suspension may contribute to the observed disinfection of *S. aureus* cells treated by the SnO_2_/Cu_2_O sample. Fig. S2 shows the Cu^2+^ concentration of Cu_2_O/SnO_2_ dispersed in 0.9% NaCl solution under visible light illumination and in dark, respectively. Cu^+^ is not stable in water and most Cu^+^ could be oxidized to Cu^2+^ under ambient environment^S8^. Before the test, nitric acid was added to oxide all remaining Cu^+^ to Cu^2+^. It demonstrated clearly that copper ion leakage happened from the Cu_2_O/SnO_2_ sample, and the Cu^2+^ concentration increased slowly with the time increase. The Cu^2+^ concentration under visible light illumination was relatively higher than that in dark, but it was still relatively low. After 35 min illumination, only ~ 0.3 mg/L of Cu^2+^ (representing the total copper ion concentration) was present in the solution. Such a low copper ion concentration was not known to have an obvious antibacterial activity^S7^. As shown in Figure 4a in the main text, even when the Cu^2+^ concentration was as high as 3 mg/L (10 times of the observed copper ion leakage concentration), the survival ratio of *S. aureus* cells could still be over 10%. Thus, the low copper ion leakage from the Cu_2_O/SnO_2_ sample could not make a major contribution to its photocatalytic disinfection and post-illumination catalytic “memory” disinfection of *S. aureus* cells.


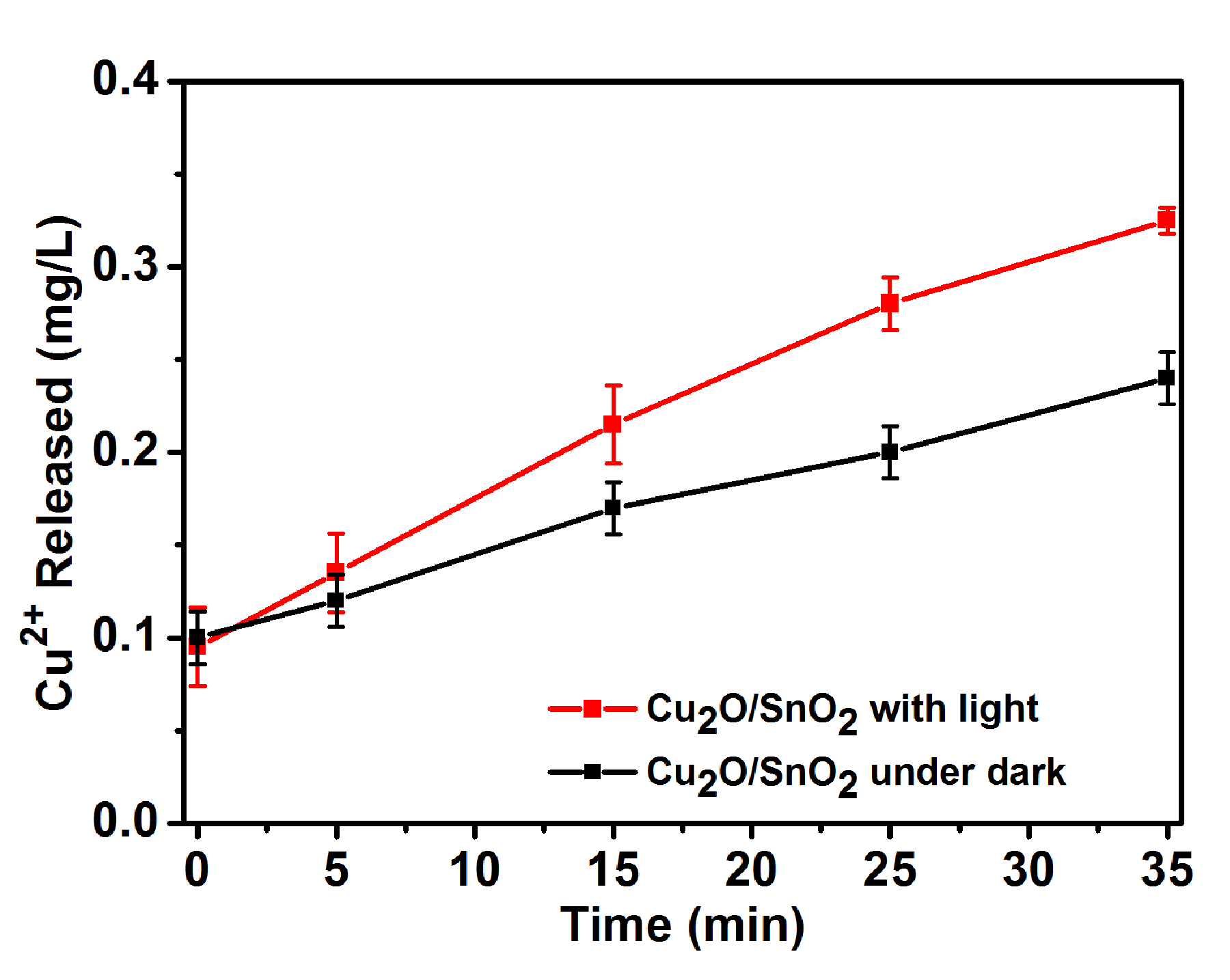


**Figure S2.** The copper ion concentration of Cu_2_O/SnO_2_ dispersed in 0.9% NaCl solution under visible light illumination and in dark, respectively.

**Methods**

**Photocatalytic Degradation of Sulfamethoxazole (SMX) under Visible Light Illumination.** Sulfamethoxazole (SMX) was used as a model organic pollutant to evaluate the photocatalytic activity of samples under visible light illumination, which is known for its strong resistance to degradation^S3,S9^. In our experiment, 0.02 g photocatalyst was dispersed in 25 mL DI water in a 150 mL glass beaker by ultrasonication for 10 min, followed by adding 25 mL of 20 mg/L aqueous SMX solution. Thus, the initial concentration of SMX in the solution was 10 mg/L, and a fixed concentration of 0.4 mg photocatalyst/mL solution was used. A 300 W xenon lamp (PLS-SXE300, Beijing PerfectLight Technology Co., Ltd., Beijing, P. R. China) was used as the light source, which has a glass filter to provide zero light intensity below 400 nm. The light intensity striking the SMX solution was at ca. 23 mW/cm^2^, as measured by a FZ-A optical Radiometer (Photoelectric Instrument Factory of Beijing Norman University, Beijing, P. R. China). At each time interval, photocatalysts were separated by centrifugation at 10,500 rpm for 5 min, and the light absorption of the clear solution was measured by the UV-2550 spectrophotometer. SnO_2_ nanoparticles and bare Cu_2_O nanocubes were also used in the photocatalytic degradation of SMX experiments for comparison purpose under the same experimental conditions. All analyses were in triplicate.

**Determination of the concentrations of copper ion.** Cupper ion (Cu^2+^) concentration was determined by using Sodium diethydlthiocabamate (DDTC) spectrophotometric method^S10^. Before the test, nitric acid was added to oxide all cupric ions (Cu^+^) to Cu^2+^. The absorbance of the solution was measured at 440 nm using the UV-2550 UV-Vis spectrophotometer to determine Cu^2+^ concentration.

**Reference**

S1. Givianrad, M. H., Saber-Tehrani, M., Aberoomand-Azar, P. & Mohagheghian, M. H-point standard additions method for simultaneous determination of sulfamethoxazole and trimethoprim in pharmaceutical formulations and biological fluids with simultaneous addition of two analytes. *Spectrochim. Acta A* **78,** 1196-1200 (2011).

S2. Chen, H., Gao, B. & Li, H. Removal of sulfamethoxazole and ciprofloxacin from aqueous solutions by graphene oxide. *J. Hazard. Mater.* **282,** 201-207 (2015).

S3. Goncalves, A. G., Orfao, J. J. & Pereira, M. Catalytic ozonation of sulphamethoxazole in the presence of carbon materials: catalytic performance and reaction pathways. *J. Hazard. Mater.* **239-240,** 167-174 (2012).

S4. Tian, Q. *et al*. Tube-like ternary α-Fe_2_O_3_@SnO_2_@Cu_2_O sandwich heterostructures: synthesis and enhanced photocatalytic properties. *ACS Appl. Mater. Interfaces* **6,** 13088-13097 (2014).

S5. Uddin, M. T. *et al*. Nanostructured SnO_2_-ZnO heterojunction photocatalysts showing enhanced photocatalytic activity for the degradation of organic dyes. *Inorg. Chem.* **51,** 7764-7473 (2012).

S6. Li, H. *et al*. Ultimate thin vertical p–n junction composed of two-dimensional layered molybdenum disulfide. *Nat. commun.* **6,** 6564 (2015).

S7. Xiong, L. *et al*. N-type Cu_2_O film for photocatalytic and photoelectrocatalytic processes: its stability and inactivation of *E. coli*. *Electrochim. Acta* **153,** 583-593 (2015).

S8. Hans, M. *et al*. Role of copper oxides in contact killing of bacteria. *Langmuir* **29,** 16160-16166 (2013).

S9. Ding, S., Niu, J., Bao, Y. & Hu, L. Evidence of superoxide radical contribution to demineralization of sulfamethoxazole by visible-light-driven Bi_2_O_3_/Bi_2_O_2_CO_3_/Sr_6_Bi_2_O_9_ photocatalyst. *J. Hazard. Mater.* **262,** 812-818 (2013).

S10. Marczenko, Z. *Spectrophotometric Determination of Elements*, E. Horwood; Wiley, **1975**.
